# Translatability and validation of non-technical skills scale for trauma (T-NOTECHS) for assessing simulated multi-professional trauma team resuscitations

**DOI:** 10.1186/s12909-019-1474-5

**Published:** 2019-01-30

**Authors:** Jussi P. Repo, Eerika Rosqvist, Seppo Lauritsalo, Juha Paloneva

**Affiliations:** 1 Department of Surgery, Central Finland Health Care District, Jyväskylä, Finland; 2 Department of Education and Science, Center of Medical Expertise, Central Finland Health Care District, Jyväskylä, Finland; 3 Department of Anesthesia and Intensive Care, Central Finland Health Care District, Jyväskylä, Finland; 4 University of Eastern Finland, Kuopio, Finland; 5 Department of Orthopedics and Traumatology, Central Finland Central Hospital, Keskussairaalantie 19, 40620 Jyväskylä, Finland

**Keywords:** Trauma team, Learning, Medical education, Teaching, Instrument, NOTECHS, Resuscitation

## Abstract

**Background:**

The 5-item non-technical skills scale for trauma (T-NOTECHS) with five response categories is developed to assess non-technical skills in trauma team resuscitations. This validated instrument assesses behavioral aspects in teamwork. Outcome instruments should undergo a robust adaptation process followed by psychometric validation to maintain their measurement properties when translated into different languages. The translatability of the T-NOTECHS into a non-Anglo-Saxon language has not been thus far unraveled. The authors aimed to assess whether the T-NOTECHS would be translatable into a non-Anglo-Saxon language and to investigate its psychometric properties for simulated multi-professional trauma team resuscitations.

**Methods:**

The T-NOTECHS (scores: 1 = poor; 5 = excellent) was translated and cross-culturally adapted into Finnish. Data was derived from 61 real hospital trauma team resuscitation simulations with 193 multi-professional participants. Floor-ceiling effects, internal consistency, and inter-rater reliability were analyzed. An exploratory factor analysis was conducted to test construct validity.

**Results:**

After pre-testing, minor changes were made to the Finnish translation of the T-NOTECHS. Mean scores of two raters were 3.76 and 4.01, respectively. The T-NOTECHS instrument showed no floor-effect either in single items or in the total score. The total score of the T-NOTECHS instrument showed a percentage of maximum scores of 1.6 and 4.9% by the Raters 1 and 2, respectively. Internal consistency (Cronbach’s alpha) was 0.70 with inter-item correlation of 0.54. The intraclass correlation coefficient was 0.54 and coefficient of repeatability 1.53. The T-NOTECHS loaded on one factor.

**Conclusions:**

The T-NOTECHS translated well into a difficult non-Anglo-Saxon language. The rigorous adaptation process used here can be recommended in the translation of observational performance assessment instruments. The translated version demonstrated fair reliability and good construct validity for assessing team performance in simulated multi-professional trauma team resuscitations. The translated T-NOTECHS instrument can be used to assess the efficacy of simulated in-situ trauma team resuscitations allowing benchmarking and international collaboration.

## Introduction

Non-technical skills are challenged during trauma resuscitation. Non-technical skills include social, cognitive and personal skills. They play an important role in the prevention of critical incidents that may lead to severe permanent disability or patient death in trauma team resuscitations [1]. In these resuscitations, non-technical skills are needed to maintain patient safety and enhancing good medical care. Good teamwork and non-technical performance are also associated with a significant decrease in disposition time [2].

During the last few years researchers and clinicians have begun to pay increased attention to non-technical skills and their effects on improving team work performance. Educating surgeons, anesthesiologists, resident surgeons and nurses is essential for making the trauma team work well together and developing their non-technical skills. Simulation-based training of non-technical skills improves these skills and thus elevates the quality of trauma resuscitation [2]. Even a short, structured two-hour trauma team simulation training course has found to be effective in improving non-technical skills among health care professionals [3]. No Nordic research that focuses on valid assessment methods and rating instruments for non-technical skills were found in a recent integrative review [4]. Therefore Nordic and international comparison of education effectiveness in simulated learning situations is challenging.

**Fig. 1:**
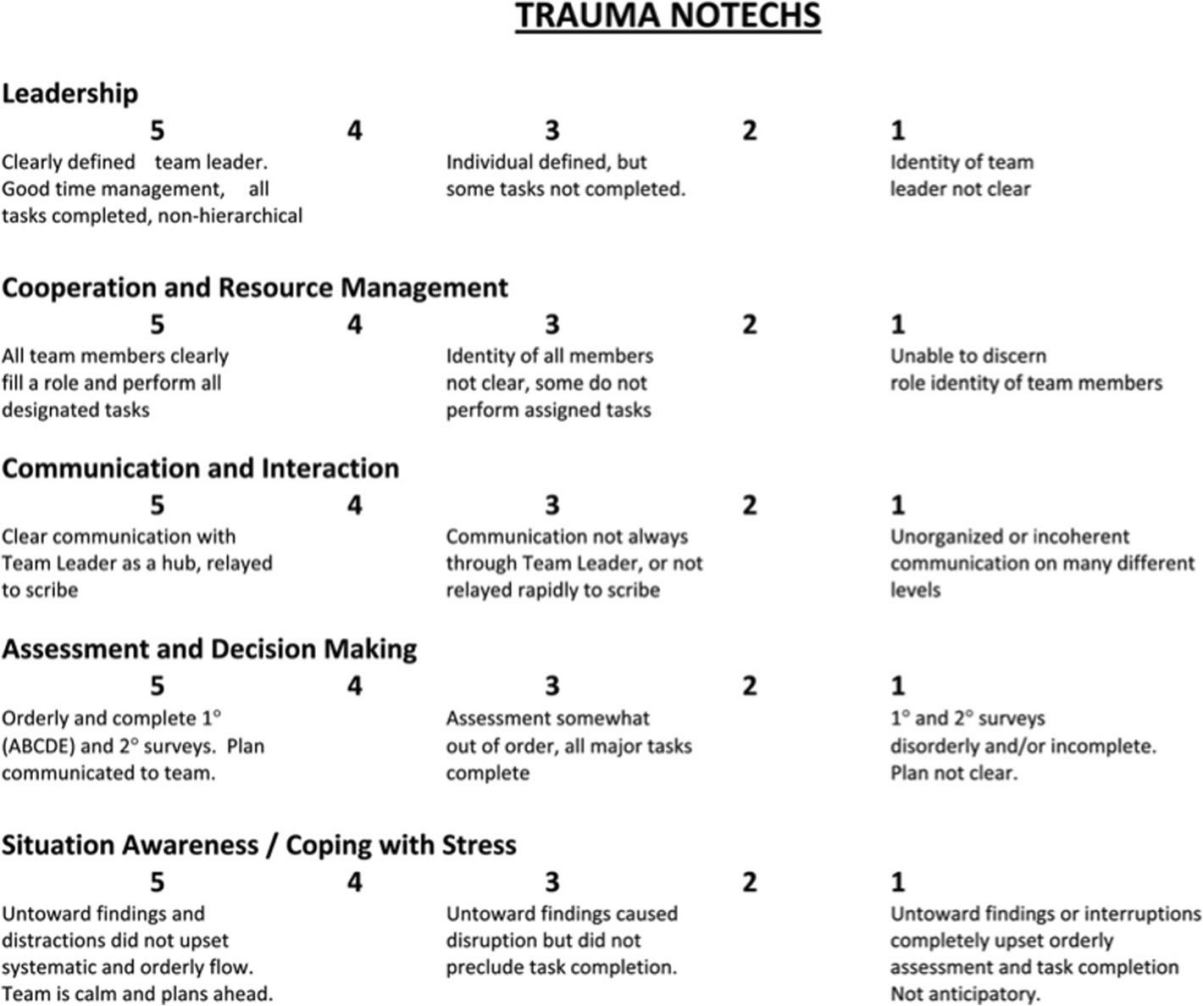
The English T-NOTECHS instrument

Instruments for rating non-technical skills (NOTECHS) are widely used and needed to assess team performance [5–8]. Steinemann et al. (2012) developed a modified version of the NOTECHS instrument (Fig. 1) to assess trauma team performance [8]. The T-NOTECHS can be used for teaching purposes, assessing learning outcomes and the teamwork skills of multi-professional trauma resuscitation teams [8]. As the language of the T-NOTECHS instrument is English, it may not be directly applicable to study populations across languages and observers due to linguistic differences and differences in the cultural or health care environment. It is of the utmost importance that the instruments used in outcome measurements are reliable and valid.

The content-related equivalence and psychometric properties of measurement instruments should be maintained when an instrument is translated into a different language. It is essential that an instrument can be easily translated into multiple foreign languages as it allows extensive, generalized research in the field of education. To fill in the methodological shortcomings for translation and adaptation of non-technical skills assessment instruments, the authors have produced robust translation and adaptation guidelines [9] based on the most pertinent literature concerning the topic of instrument translation and cross-cultural adaptation [10–21]. Knowledge of the translatability of an instrument to a foreign language is important to assure that the contents of the instrument would be comparable allowing benchmarking and comparison of international research outcomes. Description of the translation outcomes will help others to harmonize the other language versions accordingly [13]. Some parts of the instruments may not perform well in the translation process and may significantly impair the applicability of the instrument. Thus it might be optimal if international translations would be produced simultaneously during the development and validation process or the translatability of the instrument assessed [22]. The translation and adaptation process allows encounter and adjust pitfalls in the linguistic structure of the instrument.

Thus far, no instruments for assessing the non-technical skills performance of trauma resuscitation teams have been available in Finnish despite of active simulation training in Finland. The present study sought to evaluate the translatability of the T-NOTECHS into a non-Anglo-Saxon language, and to investigate the psychometric properties across in-situ full-scale multi-professional trauma team simulations. The authors hypothesized that the translated version of the T-NOTECHS would be reliable and valid for assessing team performance in simulated multi-professional trauma team resuscitations.

## Methods

### T-NOTECHS instrument

The T-NOTECHS (Fig. 1) comprises five teamwork behavioral items. These items are Leadership, Co-operation and Resource management, Communication and interaction, Assessment and decision-making, and Situation awareness/Coping with stress. Each item is scored on a 5-point scale. Item scores and the total scale score range from one to five. The lowest score of one point indicates that the team did not demonstrate the target teamwork behavior. The highest score of five points indicates flawless teamwork performance. Inter-rater reliability for video review of simulated trauma resuscitations was 0.44 by intraclass correlation coefficient (ICC) [8].

### Translation and cultural adaption of the Finnish version of the T-NOTECHS

**Fig. 2:**
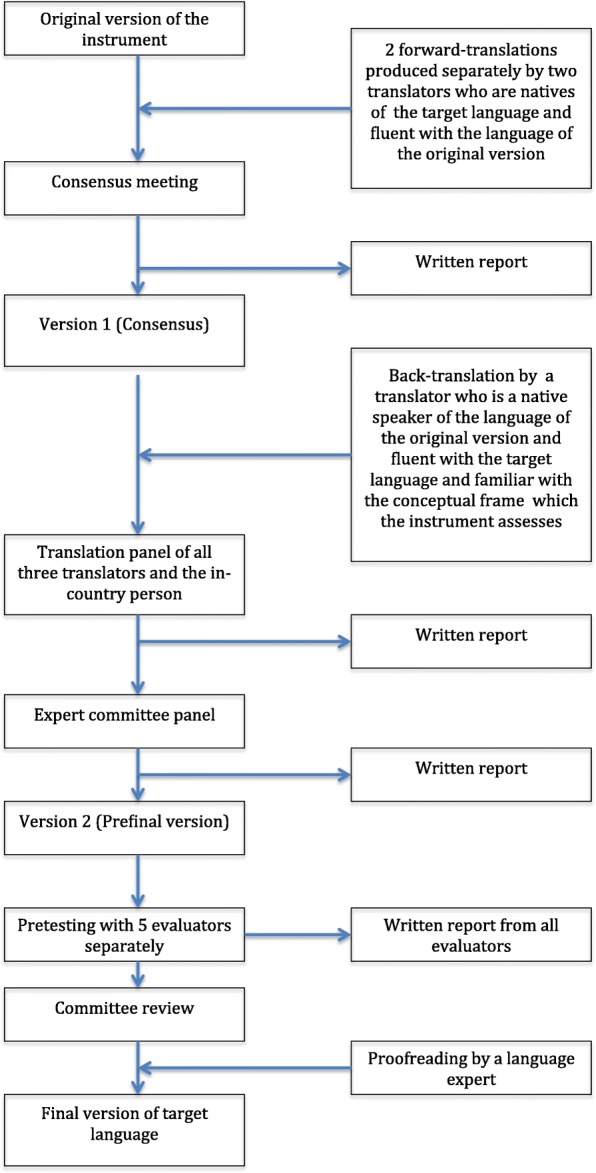
Different phases of translation process for observational instruments

The developer of the T-NOTECHS granted permission to culturally adapt and translate the questionnaire into Finnish. The translation and cross-cultural adaptation process adhered to current guidelines on the translation of non-technical skills rating scales (Fig. 2) [9]

.

Two separate translators working as healthcare professionals with Finnish as their first language and fluent in English produced the forward-translations of the T-NOTECHS from English into Finnish. The translators produced a written report and highlighted Finnish healthcare phrases and cultural features that could be misinterpreted or have more than one potential translation. A consensus of these two translations, resulting in Version 1, was made by the translators after discussion of the discrepancies between their translations.

A back-translation from Finnish into English was conducted by a native English translator fluent in Finnish. The back-translation was executed to ensure that the translated version had the same content as the original scale. The English translator had no medical background, had no knowledge of the original T-NOTECHS scale, but was familiar with Finnish culture. A back-translation panel, which included the translators and the key in-country person, reviewed the outcomes and made proposals concerning possible changes to the instrument.

An expert committee consisting of the key in-country person, project manager, an orthopedic surgeon, a specialist in anesthesiology and an anesthesia nurse compared the forward and backward translations both with each other and with the original scale and reviewed the translation reports. Key in-country person is a person who takes care of all the necessary fieldwork and participates in several of the main steps. The project manager handles the project as a whole. This phase produced a pre-final version of the Finnish T-NOTECHS (Version 2) and a written report.

The pre-final version was pre-tested by five healthcare professionals, including two trauma nurses, a specialist in emergency medicine, an emergency medical system field manager and a primary care nurse, all of whom were familiar with trauma teams. The pre-testing was conducted in two different multi-professional high fidelity in-situ full-scale trauma team simulation scenarios. Thereafter, the pre-testers were cognitively debriefed to reveal any discrepancies or problems in choosing the right item, or in understanding the items or their clarifications, and whether they would re-phrase any of the items in Finnish Version 2.

**Fig. 3:**
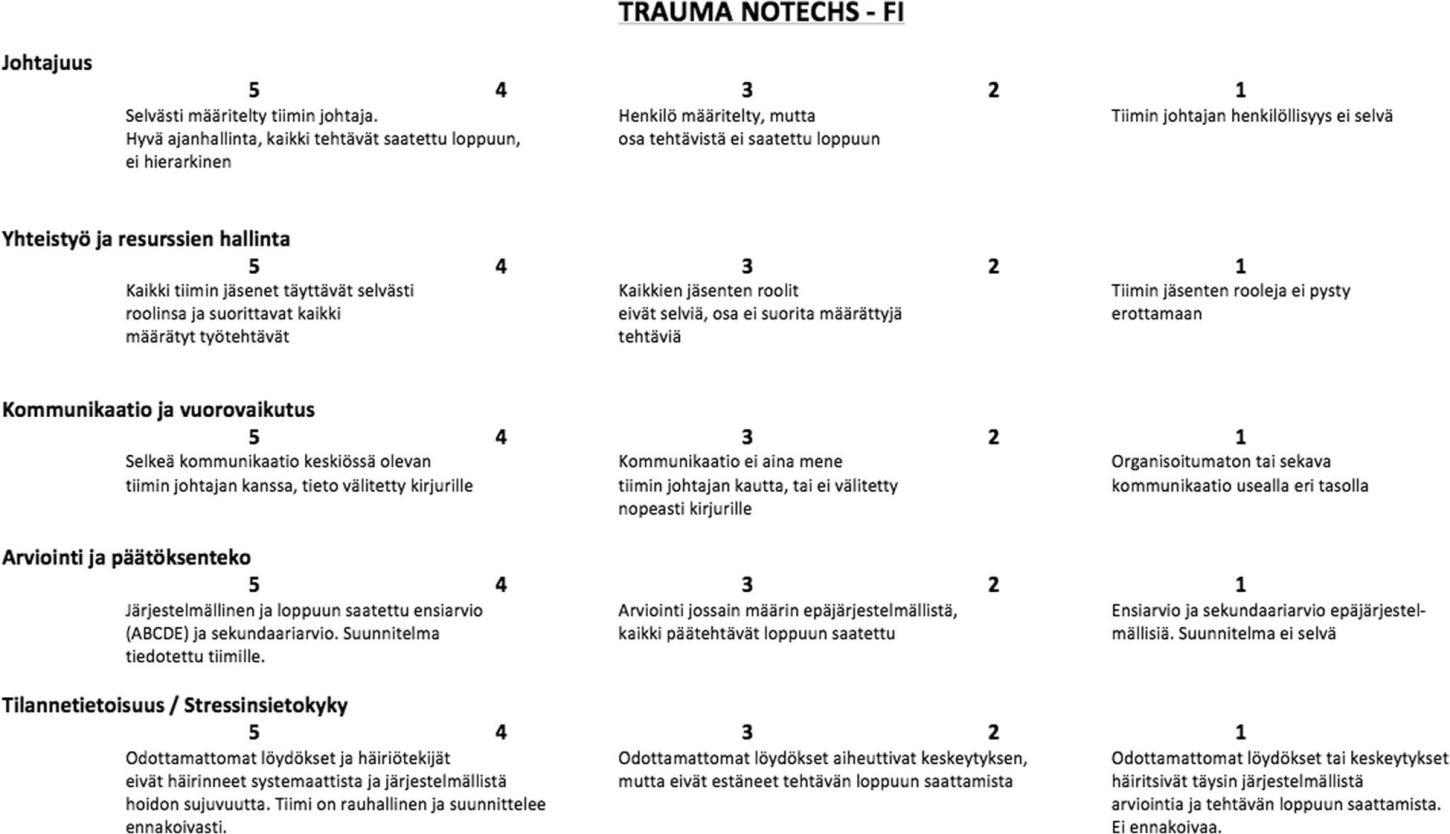
The Finnish version of the T- NOTECHS instrument

In the final phase, the multi-professional committee reviewed the outcomes of the pre-testing and the interviews. After the review, the final version of the Finnish T-NOTECHS was agreed (Fig. 3).

### Data collection

Institutional and ethical review board (*Keski-Suomen sairaanhoitopiirin Tutkimuseettinen toimikunta*) approval to conduct this study was obtained from Central Finland Health Care District. The need for consent to participate was waived by the institutional and ethical review board. The authors introduced the T-NOTECHS psychometric study settings to two raters. The raters were an anesthesiologist and a trauma nurse who had extensive experience in trauma simulations in the authors’ institution. The term “non-technical skills” was meticulously explained to them. The two raters were briefed on the concepts of rating behavior and cooperative or coordinated performance of people who are acting together as a team. Specific features and interpretation of the T-NOTECHS instrument were thoroughly discussed before the task of assessment was commenced. Each rater evaluated the trauma team resuscitation simulations independently. Both had also previous experience on using the Finnish version of the T-NOTECHS in trauma team simulations and thus had calibrated their ratings prior to the actual study data collection. The raters rated altogether 61 in-situ simulated resuscitations. The rating was performed right after the simulation had been stopped. The ratings were not further discussed with the raters or the participants. No real-life resuscitations were used for data collection in this study.

### Settings and trauma team simulation training course

The study was implemented undertaken in Central Finland Central Hospital, Jyväskylä, Finland. The Central Finland Central Hospital provides emergency trauma treatment for a catchment area of 270,000 people.

In the Central Finland Central Hospital, the trauma team includes at least a surgeon, an anesthesiologist, a radiologist, a trauma nurse and another nurse as the anesthesiologist’s working pair. Trauma team surgeons, anesthesiologists and nurses participate in simulation training once or twice a year. The structured two-hour trauma team simulation courses were conducted in Emergency Department of the Central Finland Central Hospital, and thus in a real hospital environment. The participants functioned in their real-life professional roles.

The two-hour trauma team simulation course comprised several steps. First, the training method was explained to the participants (10 min), followed by an introductory lecture (10 min), the taking up of roles (10 min), the first simulation (15 min), the first debriefing session (30 min), the second simulation (15 min) and the final debriefing session (30 min). Each of four distinct trauma simulation cases was used once (Table 1). Altogether 193 participants (Table 2) attended the trauma team simulation course at least once. Mean time (SD) to complete the first and the second simulation tasks was 12.3 (4.1) and 11.7 (2.1) minutes, respectively.

**Table 1:** Simulation case descriptions

Simulation description	Examples of trauma care procedures
Simulation I: A 36-year-old male had been stabbed twice: one stab wound in the upper right abdomen (barely bleeding) and another stab wound in the right brachium (oozing blood). Patient was pale, conscious, and drunk. GCS = 14. A. rad +. BP 108/66, HR 116, temperature line in wrist. BSs decreased on the left side. SpO2 94%, RF 24.	• Tension pneumothorax relief using a thoracocentesis or a pleural drain • Intraosseous access using a drill and intraosseous cannulation
Simulation II: A 67-year-old male had fallen 4 m from a roof to the pavement. Patient was shouting and moaning in the ER. Pelvis, abdomen, back and head were sore when examined. BSs symmetric. Temperature line in the lower leg. GCS = 10. Radial artery pulse +, BP 105/65, HR 98, RF 18, SpO2 92% with oxygen.	• FAST • Insertion of urinary catheter • Pelvic stabilization using a T-POD®
Simulation III: A 75-year-old woman had fallen from a bicycle when pushed by a car. No loss of consciousness. Patient complained of pain in shoulder, side, pelvis and ankle on the left side and had hematomas. BP 138/53, HR 93, RF 20, SpO2 96%.	• Crisis Resource Management
Simulation IV: Burn patient* from a fire in a building with burns on the face, hands, thorax, abdomen. Hair and shirt had burned off. Decreased level of consciousness. GCS 12 (3,4,5), symmetrical pupils. Smells of alcohol, 3.2 o/oo. Patient has been intubated, gags. Breath sounds symmetric with upper airway stridor. Sp02 93%, with additional oxygen 98%, RF 20. Radial artery pulse +, green cannula with Ringer 500 ml, HR 100–110. Temperature line in the lower leg. Patient had an open fracture of the femur, pelvis instability, wound in the chin and loose teeth. Extensive burns in upper torso and upper extremity (manikin masked with burned pig fat and skin).	• Escharotomy

*ER* emergency room, *GCS* Glasgow Coma Scale, *BP* Blood Pressure, *HR* heart rate, *Sp02* blood oxygen saturation level, *RF* respiratory frequency, *BS* breathing sounds, *FAST* focused assessment with sonography for trauma; *The manikin was masked with burned pig skin and fat

**Table 2:** Participants’ sociodemographic details

	*N* = 193
Gender	
Female, n (%)	108 (55)
Male, n (%)	85 (45)
Age, years, mean (SD)	37 (9.2)
Occupation, n (%)	
Anesthesiologist*	38 (20)
Surgeon*	38 (20)
Pediatrician*	3 (2)
Emergency medicine resident	22 (11)
Nurse	83 (43)
Nurse student	9 (4)
Length of experience in present job years, mean (SD)	7.4 (7.5)
Participation in trauma team simulations, times, mean (SD)	5 (4)
Participation in real life trauma team resuscitations, times, mean (SD)#	11 (19)

*specialist or resident; # calculated for 165 participants – 30 participants had no previous experience

The simulation training main instructor (SL) was a senior anesthesiologist and an intensive care physician. He is specialized as a medical educator and is a specialist in emergency medicine in the Finnish Medical Association. He also had an Advanced Trauma Life Support training provider, and had participated in the Finnish basic and advanced courses for simulation instructors and in the European Trauma Course. Two nurse teachers (anesthesia nurses) alternated as his working pair and piloted the simulation. A computer-based adult patient simulator was used (HAL S3201, Gaumard, Nordic Simulators Oy).

### Statistics

Data are presented as means with standard deviations (SD), medians with interquartile ranges (IQR), 95% confidence intervals (95% CI), or as counts with percentages. Predefined hypotheses were set based on the existing literature (Table 3). Floor and ceiling values were calculated by dividing the number of assessments receiving maximum or minimum scores, respectively, by the total number of assessments. Internal consistency was estimated by calculating Cronbach’s alpha. Relative reliability, using an ICC, was calculated using a two-way random-effect model with absolute agreement. The ICC value was classified according to Cicchetti et al. as poor (< .40), fair (.40–.59), good (.60–.74) or excellent (.75–1.00) [23]. Absolute reliability was assessed with a coefficient of repeatability (CR), expressing the absolute differences between paired observations.

**Table 3:** Predefined hypotheses

Concept	Rejected/Confirmed
Floor and ceiling values are ≤15%	Confirmed
Internal consistency .70–.90	Confirmed
Inter-rater reliability > .40	Confirmed
Loads on one factor	Confirmed

To investigate construct validity of the T-NOTECHS, statistical analyses were used to identify the factor loadings. A principal component exploratory factor analysis (EFA) was conducted using the varimax rotation with Kaiser normalization. The data for this analysis was obtained by adding the scores of the two observers as a one series of evaluations to allow performing factor analysis for a larger data set. If the eigenvalue is over one, the factor is considered confirmed. The authors hypothesized that the T-NOTECHS would load on one factor that would indicate that the instrument measures one latent trait (non-technical skills).

Statistical analyses were performed using SPSS 24.0 (SPSS Inc., Chicago, IL, USA). The research report adhered to the STROBE checklist [24]. Study sample size was chosen according to the COSMIN checklist for psychometric studies to represent sample size that is considered good [25].

## Results

### Translation and cultural adaptation

Minor translation differences were encountered between the two forward translations and discussed by the translators in phase two. The back-translation panel review revealed no major linguistic problems between the back-translation and the original English version. The pre-final Finnish language version was accepted after comparing the content of the original and backward translated version. Three of the five pre-testers declared the scale understandable and easy to fill in. The two remaining pre-testers found the words used to describe leadership problematic. The committee decided that item 3, “Communication and interaction”, needed refinement: the word “hub” was deemed confusing in Finnish and thus omitted, and the sentence rephrased so that its content remained unchanged. Minor rephrasing was required in the 3rd response category of the Likert scale for the item “Assessment and decision making”. All the committee members approved these minor changes, which were thought to improve the external validity of the Finnish version of the T-NOTECHS and all the pre-testers considered the items of the Finnish version of the T-NOTECHS to accurately reflect the original, thereby confirming the face validity of the instrument.

### Reliability and validity

#### Floor-ceiling effect

The T-NOTECHS instrument showed no floor-effect (lowest possible score) either in single items or in the total score (Table 4). A ceiling effect (over 15% of scores) was noted for items 1 and 5 in both raters. Analyses revealed a ceiling effect for all the scores of Rater 2. However, the total score of the T-NOTECHS instrument showed no ceiling effect, as the percentage of maximum scores given by Raters 1 and 2 was 1.6 and 4.9%, respectively.

**Table 4:** Score parameters for the T-NOTECHS obtained from the performance of 61 trauma teams

Domain	Mean, (SD)*		Completion rate (%)		Minimum score, n of		Maximum score, n of (%)		Corrected item-total correlation	
	Rater 1	Rater 2	Rater 1	Rater 2	Rater 1	Rater 2	Rater 1	Rater 2	Rater 1	Rater 2
Leadership	3.79 (0.88)	4.07 (0.77)	100	100	0	0	12 (20)	19 (31)	.68	.80
Cooperation and Resource management	3.89 (0.61)	4.05 (0.56)	100	100	0	0	8 (13)	11 (18)	.67	.68
Communication and interaction	3.74 (0.68)	3.92 (0.67)	100	100	0	0	7 (12)	11 (18)	.58	.75
Assessment and decision making	3.59 (0.62)	3.97 (0.60)	100	100	0	0	2 (3)	10 (16)	.84	.69
Situation awareness/coping with stress	3.79 (0.71)	4.41 (0.64)	100	100	0	0	9 (15)	30 (49)	.73	.67

*Standard Deviation; Rating: 5 indicates flawless teamwork, 1 indicates the team did not demonstrate this teamwork behavior

#### Internal consistency and item analysis

Cronbach’s alpha for the T-NOTECHS total score revealed an acceptable internal consistency of 0.70. The corrected item-total correlation varied between 0.58 and 0.84 in Rater 1, and 0.67 and 0.80 in Rater 2 (Table 4). The highest corrected item correlation was found for item 4 in Rater 1 and item 1 in Rater 2. The median (IQR) of all the items combined was 0.68 (0.67) for Rater 1 and 0.69 (0.68) for Rater 2.

#### Inter-rater reliability

The mean scores were 3.76 (0.71) for Rater 1 and 4.01 (0.67) for Rater 2. The difference between the two raters in their mean scores for the T-NOTECHS instrument was 0.25 points. The ICC was 0.54 (95% CI, 0.34–0.70) and the CR was 1.53 (95% CI, 1.29–1.94). All predefined hypotheses concerning instrument reliability were met.

#### Factor analysis

**Fig. 4:**
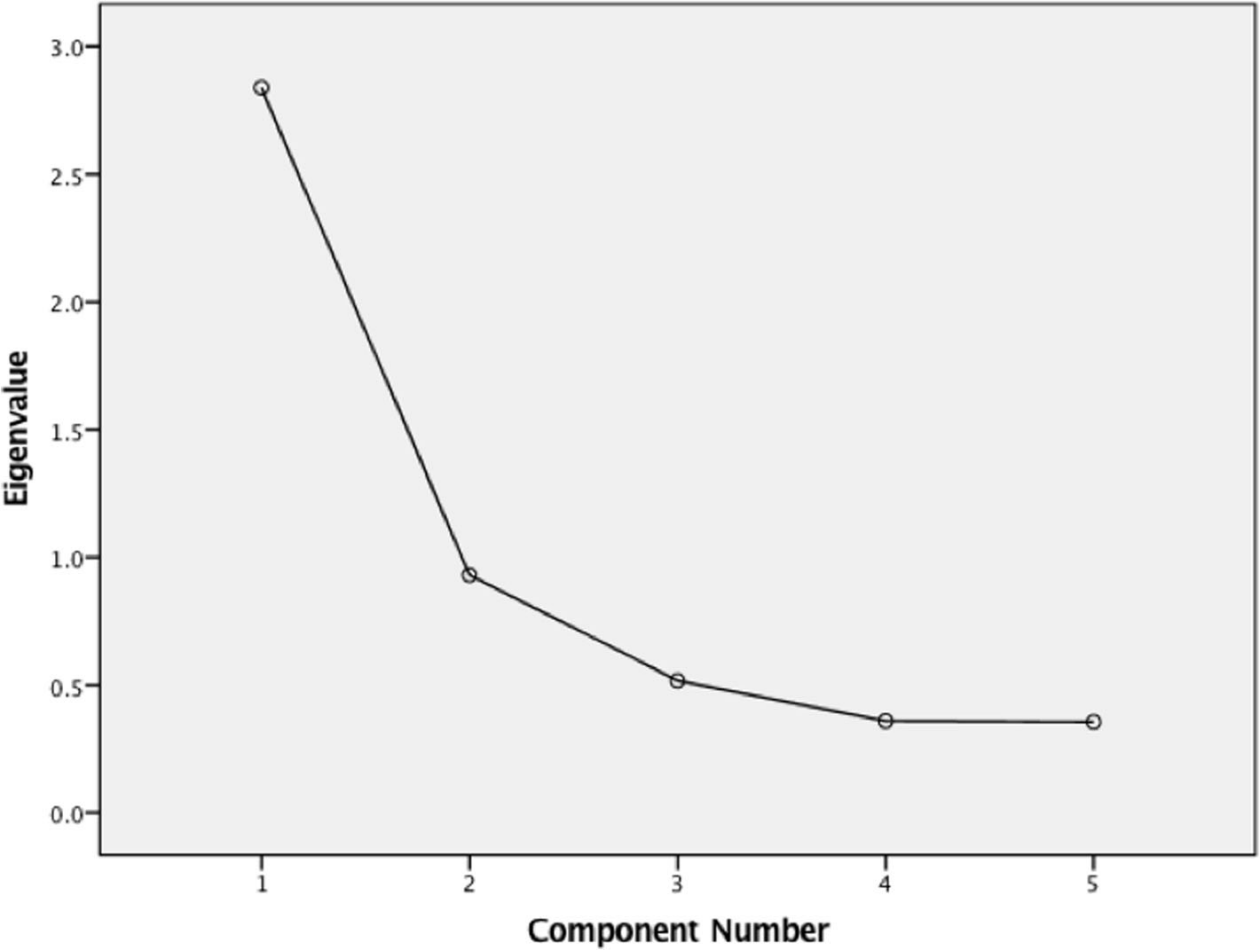
A scree plot illustrating eigenvalues and components in the exploratory factor analysis of the T- NOTECHS

In the EFA, the T-NOTECHS loaded on one factor. The first component had an eigenvalue of 2.84 indicating a strong factor and the second factor had an eigenvalue of 0.93 (Fig. 4). Four items loaded on the first factor explaining 56.8% of the total variance (Table 5). The item “Cooperation and resource management” loaded on the second factor explaining 18.5% of the total variance. Thus the hypothesis of one factor instrument was accepted.

**Table 5:** Factor loadings of the exploratory factor analysis

*Item description*	*Factor 1*
Leadership	0.383
Communication and interaction	0.822
Assessment and decision making	0.753
Situation awareness/Coping with stress	0.847
Cooperation and resource management	0.855

## Discussion

This study confirmed that the T-NOTECHS instrument is translatable into a difficult non-Anglo-Saxon language, Finnish, that is a synthetic language, includes consonant gradation and consonant clusters, and lacks grammatical gender. The present psychometric testing of the non-Anglo-Saxon version of the T-NOTECHS instrument showed a level of reliability comparable to that of the original English version and gave further insight of its good construct validity for assessing team performance in simulated multi-professional trauma team resuscitations. All predefined hypotheses of reliability and validity were met. The T-NOTECHS instrument seemed to measure one latent construct (non-technical skills).

The authors started conducting computerized patient simulator-based multi-professional trauma team simulation training in the Central Finland Central Hospital in 2009. The authors’ Center of Medical Expertise is accredited by the Network of Accredited Clinical Skills Centres in Europe (NASCE). In the authors’ institution, trauma team simulation training is used as a regular teaching and learning method to improve and maintain trauma team performance. This structured in situ two-hour multi-professional simulation training course has been proved to be effective in improving NTS among 90 trauma teams with altogether 430 participants [2].

Specifically, focus areas of trauma team simulation training are to enhance the effectiveness of team performance. Skill development takes place through improving team members’ knowledge of the trauma resuscitation guidelines, problem identification, assessment together with decision making, situation awareness/coping with stress, teamwork/cooperation and resource management, communication and interaction, time management, being under authority and confidence in one’s role in a trauma team. Simulation tasks also stress the importance of team leaders’ understanding of their leadership, the distribution of workloads and conflict resolution.

There was felt to be an urgent need to assess the efficacy of the training program with appropriate assessment methods. However, no non-technical skills rating instruments for assessing trauma team performance were available in Finnish. Hence, the T- NOTECHS instrument was translated into Finnish and adapted to the Finnish culture and its measurement properties were investigated.

### Translation and cultural adaptation

The fact that most of the non-technical skills rating scales have been developed in English means that they are unlikely to be directly applicable to different language study populations and observers. It is thus of paramount importance that instruments used to provide outcome measurements retain their original meaning and content. To guarantee the comparability and validity of study outcomes, it is essential that the psychometric properties of measurement instruments remain unchanged when translated into another language. Before this study, no specific translation and cultural adaptation guidelines were available for the translation of non-technical skills rating instruments. To fill this methodological gap, the present authors first established solid and structured guidelines for the translation and adaptation of non-technical skills rating scales for use different languages and cultures. This facilitated the translation process, as only minor linguistic changes were subsequently required. However, no other cross-culturally adapted versions of the T-NOTECHS scale with which to compare and harmonize the translation results of the present study existed. Future translations should use similar structured translation process as described here as it proved applicable for a difficult non-Anglo-Saxon language.

### Reliability and validity

The present study found no floor or ceiling effects at the cut off of 15% in the T-NOTECHS instument. However, the items “Leadership” and “Situation awareness/coping with stress” showed ceiling values of 20 and 15 in Rater one, and 19 and 30 in Rater two. These results should be taken into consideration when evaluating the scores of the T-NOTECHS as they could have a notable impact on the total score.

Cronbach’s alpha of 0.70 demonstrated adequate internal consistency for the Finnish T-NOTECHS. The outcomes of this analysis indicate minimal item repetition in the T- NOTECHS instrument. Thus the alpha value for the T-NOTECHS can be considered acceptable for assessing trauma team performance.

The average measures ICC indicates inter-rater reliability. In the present instance, it was hypothesized that the ICC value would exceed 0.40. The actual ICC value of 0.54 confirmed the hypothesis, and thus indicated a fair level of reliability among the 61 real-time trauma team simulation resuscitations. Compared to the present findings, Steinemann et al. reported a lower relative reliability of 0.44 for the T-NOTECHS instrument [7]. Steinemann used three raters who rated a total of 33 simulation cases in real time (immediately after resuscitation). These differences between the original study of Steinemann et al. and the present study could be due to the different trauma team resuscitation settings or to the numbers of simulations or raters. Furthermore, in the present study, CR was used to estimate the absolute reliability of the scale. The CR refers to a cut-off value where the absolute difference between two measurements can be estimated with a probability of 95%. In the present study, the absolute reliability of the T-NOTECHS instrument in the in-situ trauma team resuscitations was 1.53. The CR may yield a more accurate assessment of the absolute reliability of a scale than the standard error of measurement as it presents a true difference between the raters.

Steinemann et al. stressed the importance of rater training in the original T-NOTECHS validation paper [7]. They presented their raters with trauma teamwork examples. In the authors’ experience, it might be advisable to use at least two raters to minimize the risk of bias. It is important that raters are carefully briefed in the use of the T-NOTECHS instrument. This may yield more accurate scores.

The exploratory factor analysis showed that the T-NOTECHS loaded on one factors (eigenvalue over one). This refers to that the scores obtained from the T-NOTECHS are suitable to be used as an index score. This analysis gives insight of the good construct validity of the T-NOTECHS instrument.

### Strengths and limitations

To the authors’ knowledge, the present study used the largest number of trauma team resuscitation simulations thus far to estimate the reliability of the T-NOTECHS instrument. According to the COSMIN checklist, the sample size used was good [25]. Further strengths of the present study were that the raters were meticulously trained to evaluate team performance, the assessment was implemented in a real hospital environment, a large number of participants attended the simulations, and several different simulations were assessed, allowing greater generalizability of the results. The authors also described the study phases in detail and used the structured COSMIN checklist in reporting the outcome. A notable limitation of this study was the absence of other comparable T-NOTECHS outcome measures for validity assessment. However, good face validity in the T-NOTECHS was noted during the translation phases and factor analysis was conducted to construct validity testing. The results of this study can be applied to trauma team resuscitations. Further studies should include psychometric testing using Rasch Measurement analytic techniques to give more insight or the construct validity of the T-NOTECHS, assessments of its reliability in actual trauma team resuscitations, for high-stakes assessment and further convergent and construct validity testing using other reference measures for correlation comparison. Future studies could also demonstrate the ability of the T-NOTECHS instrument to discriminate between high and low performances, either in real clinical practice or using performance during simulation.

## Conclusions

The authors conclude that the T-NOTECHS translated well into a difficult non-Anglo-Saxon language. The rigorous adaptation process used in this paper can be recommended in the translation of non-technical skills instruments. Psychometric testing of the translated version provided evidence of fair inter-rater reliability and construct validity for the T-NOTECHS for assessing team performance in simulated real-time hospital multi-professional trauma team resuscitations. The T- NOTECHS is a suitable instrument for assessing non-technical skills in performance in simulated trauma team resuscitations that include surgeons, anesthesiologists and/or resident physicians. The translated T-NOTECHS instrument can be used to assess the efficacy of simulated in-situ trauma team resuscitations allowing benchmarking and international collaboration.

## Abbreviations

COSMIN: COnsensus-based Standards for the selection of health Measurement INstruments
CR: Coefficient of repeatability
EFA: Exploratory factor analysis
ICC: Intraclass correlation coefficient
IQR: Interquartile range
SD: Standard deviation
STROBE: STrengthening the Reporting of OBservational studies in Epidemiology
T-NOTECHS: non-technical skills scale for trauma
